# Radiation induces acid tolerance of *Clostridium tyrobutyricum* and enhances bioproduction of butyric acid through a metabolic switch

**DOI:** 10.1186/1754-6834-7-22

**Published:** 2014-02-18

**Authors:** Xiang Zhou, Xi-Hong Lu, Xue-Hu Li, Zhi-Jun Xin, Jia-Rong Xie, Mei-Rong Zhao, Liang Wang, Wen-Yue Du, Jian-Ping Liang

**Affiliations:** 1 Institute of Modern Physics, Chinese Academy of Sciences, 509 Nanchang Road, Lanzhou, Gansu 730000, PR China; 2 China Pharmaceutical University, #24 Tongjiaxiang, Nanjing 210009, PR China

**Keywords:** Acid inhibition, Butyrate, *Clostridium tyrobutyricum*, ^12^C^6+^ heavy ion, Fermentation, Irradiation

## Abstract

**Background:**

Butyric acid as a renewable resource has become an increasingly attractive alternative to petroleum-based fuels. *Clostridium tyrobutyricum* ATCC 25755^T^ is well documented as a fermentation strain for the production of acids. However, it has been reported that butyrate inhibits its growth, and the accumulation of acetate also inhibits biomass synthesis, making production of butyric acid from conventional fermentation processes economically challenging. The present study aimed to identify whether irradiation of *C. tyrobutyricum* cells makes them more tolerant to butyric acid inhibition and increases the production of butyrate compared with wild type.

**Results:**

In this work, the fermentation kinetics of *C. tyrobutyricum* cultures after being classically adapted for growth at 3.6, 7.2 and 10.8 g·L^-1^ equivalents were studied. The results showed that, regardless of the irradiation used, there was a gradual inhibition of cell growth at butyric acid concentrations above 10.8 g·L^-1^, with no growth observed at butyric acid concentrations above 3.6 g·L^-1^ for the wild-type strain during the first 54 h of fermentation. The sodium dodecyl sulfate polyacrylamide gel electrophoresis also showed significantly different expression levels of proteins with molecular mass around the wild-type and irradiated strains. The results showed that the proportion of proteins with molecular weights of 85 and 106 kDa was much higher for the irradiated strains. The specific growth rate decreased by 50% (from 0.42 to 0.21 h^-1^) and the final concentration of butyrate increased by 68% (from 22.7 to 33.4 g·L^-1^) for the strain irradiated at 114 AMeV and 40 Gy compared with the wild-type strains.

**Conclusions:**

This study demonstrates that butyric acid production from glucose can be significantly improved and enhanced by using ^12^C^6+^ heavy ion-irradiated *C. tyrobutyricum*. The approach is economical, making it competitive compared with similar fermentation processes. It may prove useful as a first step in a combined method employing long-term continuous fermentation of acid-production processes.

## Background

The trends of globalization and population expansion, in addition to technological advances, have led to skyrocketing energy demands worldwide [1]. The annual oil consumption, currently at around 32 billion barrels, continues to rise at the rate of population growth - about 1% a year (chart by Morgan Downey). As oil prices remain high, there are once again murmurs of anticipated doom from various quarters. This is reflected in the transportation industry where increased demand and a limited supply of fuel have resulted in vastly inflated prices. While more oil deposits will likely be identified in the future, the fact remains that oil is a non-renewable resource that will eventually run out. Thus, the use of various kinds of renewable energy sources is of interest throughout the world.

The consumers’ preference is for bio-based natural ingredients as additives for increasing fruit fragrance and aromatic compounds for production of perfumes [2-6]. Butyric acid is a short-chain fatty acid produced from sugars by *Clostridia* species such as *Clostridium tyrobutyricum*, *C. beijerinckii*, *C. butyricum*, *C. populeti* sp*.*, *C. thermobutyricum* sp. nov. and *C. acetobutyricum*. Currently, the most promising microorganism used for the bioproduction of butyric acid is *C. tyrobutyricum* ATCC 25755^T^[7-10]. The microorganism is a Gram-positive, rod-shaped, spore-forming, obligate anaerobic bacterium. Its main fermentation products are butyric acid, acetate, n-butanol, acetic acid, hydrogen and carbon dioxide from various carbohydrates including glucose, xylose, fructose, disaccharides, sucrose and lactose [11-16]. This strain is known to produce CO_2_ and hydrogen and grows well at pH 6.0 and 37°C [17-19]. It is known that phosphotransacetylase *Clostridium* can utilize various polymeric substrates, including cellulose and galactan, with very little production of unnecessary materials [20-22]. In addition to its industrial use as a chemical feedstock, butyric acid has recently drawn strong interest as a precursor for biobutanol production via bioconversion [23,24]. Biobutanol (108.46 British thermal units per gallon) is a next-generation biofuel, primarily as a petroleum derivative, because of its low vapor pressure, high energy content and similar energy content to gasoline [25-28]. Butyrate can also be bioconverted to butanol by the fermentation action of certain strains of bacteria [29-31]. Moreover, biobutanol, like other biofuels, has many advantages, including that it is more economical, renewable, environmentally friendly and carbon neutral [32-34]. Normally, butyric acid is produced through the oxosynthesis of butyraldehyde from propylene [35-37]. However, the chemical synthesis of butyric acid is not attractive or sustainable because the raw materials are obtained from fossil fuels [38,39].

As such, butyrate fermentation is an alternative method for producing butyrate [40-44]. *C. tyrobutyricum* ATCC 25755^T^ has been extensively studied for its ability to produce butyric acid and hydrogen [45-50]. There has been increasing interest in the production of butyric acid from agricultural commodities and processing wastes using *C. tyrobutyricum* ATCC 25755 [51-53]. The metabolic pathway for butyric acid fermentation of glucose, pentose, xylose and hexose in acidogenic *C. tyrobutyricum* ATCC 25755 has several possible end-products, including butyrate, with acetate, CO_2_, H_2_ and lactate as its main fermentation byproducts. Two analogous pathways lead to the formation of acetate and butyrate, with acetyl-coenzyme A (CoA) and butyryl-CoA functioning as key intermediates, respectively. First, acetyl phosphate and butyryl phosphate are produced from their CoA derivatives, catalyzed by phosphotransacetylase (PTA) and phosphotransbutyrylase (PTB), respectively. Then these acyl phosphates are converted to acetate and butyrate, catalyzed by acetate kinase (AK) and butyrate kinase (BK), respectively [18,48,54]. However, conventional butyric acid fermentation biotechnology is not yet economically competitive. For instance, a number of studies have shown that the butyrate yield is only 0.8 to 1.1 mol·mol^-1^ of fermented glucose, pentose, xylose and hexose, and the acetate yield is only 0.32 to 0.42 mol·mol^-1^[4,24,55-58].

Strain improvement by mutagenesis and selection is a highly developed technique that plays a central role in the commercial development of microbial fermentation processes. The Heavy Ion Research Facility in Lanzhou (HIRFL) is committed to finding solutions to real problems through basic research. HIRFL comprises a superconducting Electron Cyclotron Resonance ion source, the 1.7 m Sector Focused Cyclotron (K = 69), the large Sector-Separated Cyclotron (K = 450), the Cooler-Storage Main Ring (CSRm) and the Cooler-Storage Experimental Ring of the newly built Cooler-Storage Ring, the radioactive ion beam lines (Radioactive Isotope Beam Line in Lanzhou - RIBLL1 and RIBLL2) and experimental terminals. It is capable of providing ion beams from protons to uranium with energies of up to 2,800 MeV u^-1^ and 1,000 MeV u^-1^ for protons and heavy ions, respectively. Mutagenic procedures can be carried out by varying the type of mutagen and dose to obtain mutant types that may be screened for improved activity [59-61]. At present, not only have mutagenic sources such as neutrons, UV light, *α*-rays, *γ*-rays and lasers been developed and successfully employed to acquire various sorts of valuable strains, but novel sources are still under development with the aim to obtain a wider mutation spectrum and a higher ratio of mutation [62-67]. In recent years, the term irradiation technology has also been used to refer to novel techniques such as ^12^C^6+^-ion irradiation. Specifically, ^12^C^6+^-ion beam irradiation is a type of high linear energy transfer irradiation that is used to bombard the target with higher energy than can be achieved using different linear energy transfer forms of irradiation [68-71]. However, linear energy transfer, energy, and radiation dose may play a more fundamental role in regulating the synthesis and secretion of microbial biofuels.

To the best of our knowledge, measurements of the butyric acid levels produced by *C. tyrobutyricum* ATCC 25755 strains as a result of combined ^12^C^6+^-ion beam radiation and fermentation have not been reported. The main objective of this study was to evaluate and characterize these *C. tyrobutyricum* mutants for their ability to produce butyric acid from glucose as a carbon source. In this work, the kinetics of cell growth and butyrate/butyric production in culture fermentation by mutant and wild-type *C. tyrobutyricum* were studied and compared.

## Results and discussion

### Cell survival after irradiation

Investigation of radiation-induced cell growth and death, defined as the time period required for a complete loss of the proliferation capacity or exaltation of the proliferation capacity, is one of the most commonly and reliably used methods to study radiation effects on cells. For the irradiation experiments, our laboratory verified that the 3-(4,5-dimethylthiazol-2-yl)-2,5-diphenyltetrazolium bromide (MTT) readings were proportional to the number of cells *in vitro*, at least in the phase of exponential growth (data not shown). ^12^C^6+^ ion irradiation at high energy usually results in the death of the vast majority of cells. The fraction of cell death in the lag phase after irradiation and changes in doubling-time can be measured by assaying at various time points after irradiation. Because our assay was not only a single-point determination of survival, information about growth performance could also be acquired easily. The survival curve was drawn on a natural logarithmic scale of the survival fraction versus different physical parameters.

**Figure 1:**
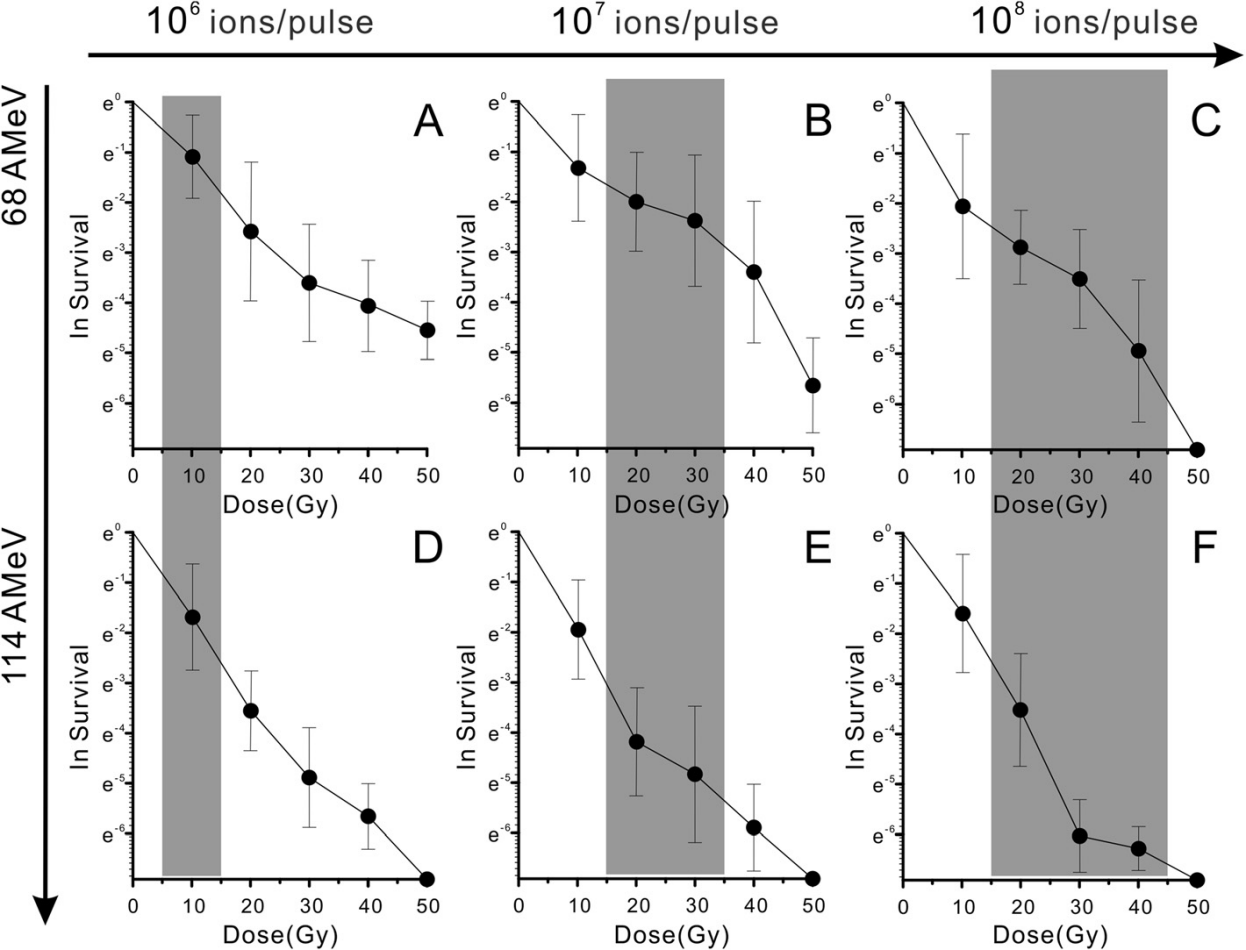
**The effect of **^**12**^**C**^**6+**^**-ion irradiation on the survival of *Clostridium tyrobutyricum *ATCC 25755.** The cells were irradiated 20 h after seeding and the extent of survival was determined by MTT assay. Survival data is plotted against the irradiation levels. **(A-C) **^12^C^6+^-ions were accelerated up to 68 AMeV, and their ions/pulse ranged from 10^6^ to 10^8^, at a dose rate of 10 to 50 Gy. **(D-F) **^12^C^6+^-ions were accelerated up to 114 AMeV, and their ions/pulse ranged from 10^6^ to 10^8^, at a dose rate of 10 to 50 Gy. Cells with low metabolic activity and slow proliferation or cells that cease to proliferate were excluded from the assay by washing and trypsinization when the plating was done after irradiation.

*C. tyrobutyricum* 25755 cells were irradiated 20 h after seeding. The strains with the lowest metabolic activity and slowest proliferation or cells that ceased to proliferate were excluded from the assay by washing and trypsinization when the plating was done after irradiation. The survival fraction as obtained from Equation (1) was compared with a representative set of experimental data. Figure 1 shows a comparison of the survival curves after ^12^C^6+^-ion irradiation at different beam energies for the various strains of *C. tyrobutyricum* ATCC 25755. The results of the MTT assay are plotted against the irradiation dose (10 to 50 Gy) at 68 AMeV of energy and 10^6^ to 10^8^ ions · pulse^-1^ levels, which were *e*^*0*^ → *e*^*-4.5*^ for Figure 1A, *e*^*0*^ → *e*^*-5.8*^ for Figure 1B and *e*^*0*^ → *0* for Figure 1C. Figure 1D-F shows the cellular survival data from the results of the MTT assay against the irradiation dose (10 to 50 Gy) at 114 AMeV of energy and 10^6^ to 10^8^ of ions·pulse^-1^ levels, which were *e*^*0*^ → *0*. In general, sufficient agreement between the calculations and experimental data was obtained. For the strains treated at 68 AMeV, the equation underestimated the effectiveness of the dose, whereas for the cells irradiated at high energies (114 AMeV), the result was overestimated. The maximal deviation, derived from the ratio of calculated to measured doses for a given effect level, was 15%. The survival fraction of the strains strongly depended on the particular physical characteristics of the ^12^C^6+^-ion beam, as determined by the energy, dose and ions · pulse^-1^ levels of the particles under consideration (Figure 1). Obviously, the survival fraction decreased with increasing carbon ion energy. As expected, the survival logarithmic of the assays showed the same characteristics: the survival depended on the energy, ions·pulse^-1^ and dose of ^12^C^6+^-ion irradiation. The increase of one physical parameter at a time led to a decrease in survival rate. Very limited survival (*e*^*-3.5*^ → *e*^*-6.5*^) was obtained when the ^12^C^6+^-ion was irradiated using 114 AMeV of energy, a 20 to 40 Gy dose and 10^6^ to 10^8^ ions·pulse^-1^.

Many cell types are characterized by regular cell division every 12 to 24 h. Because of the power of exponential growth, a single cell can produce thousands of daughter cells within approximately 9 to 12 normal division cycles, that is, a few days. After irradiation, the survivors may then be composed of some mutants. A very small percentage of the *C. tyrobutyricum* ATCC 25755 survivors may show an improved ability to produce butyrate.

### The effects of butyric acid on cell growth following irradiation

**Figure 2:**
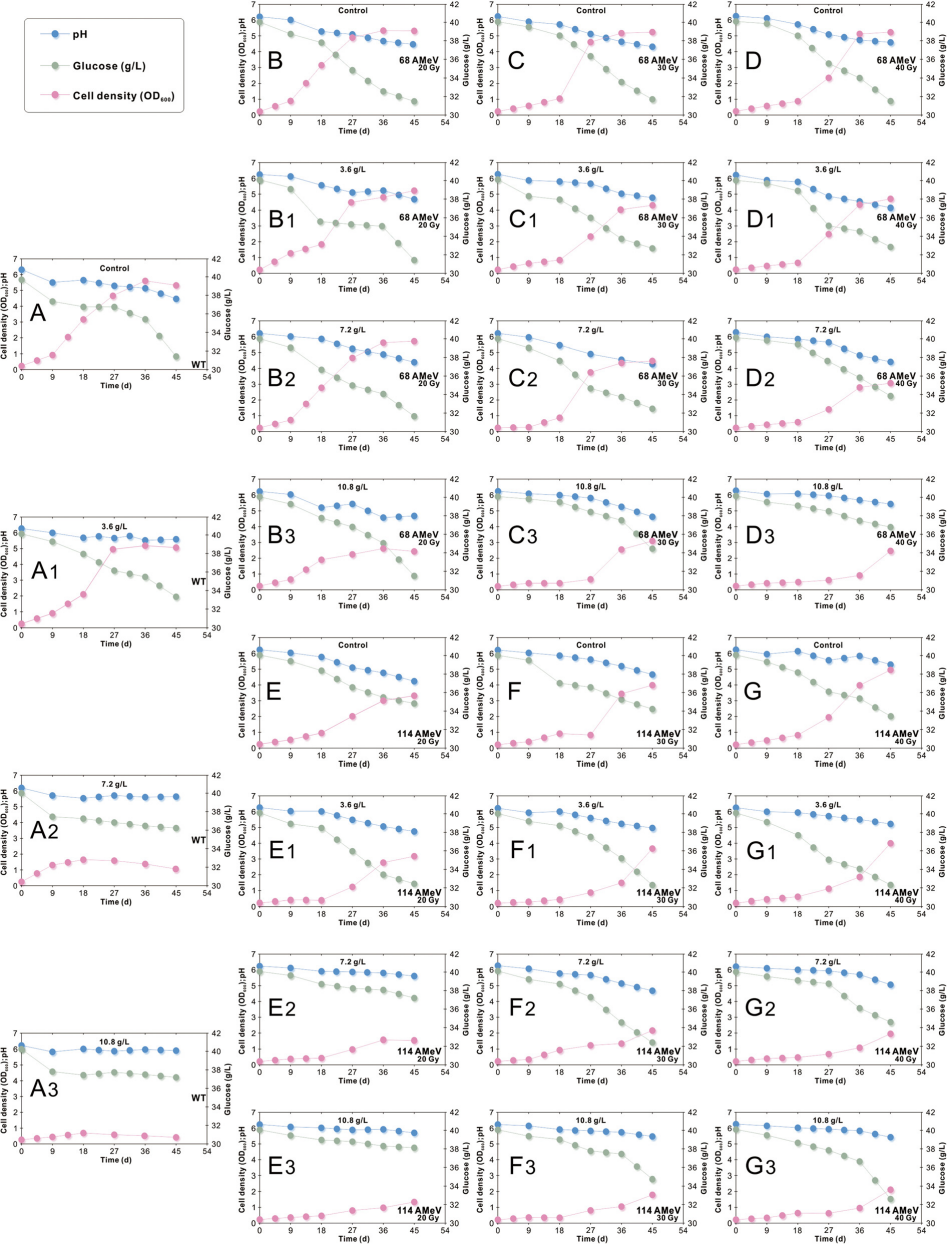
**Time-course of the activities of wild-type and irradiated cells as a function of added butyric acid concentrations during the first 54 h of fermentation. (A)** Cell growth of the wild-type strains, unaffected by the addition of butyrate (control cultures). **(B-D)** Cell growth of the irradiated strains (energy 68 AMeV and doses of 20, 30 and 40 Gy), unaffected by the addition of butyrate (control cultures). **(E,F)** Cell growth of the irradiated strains (energy 114 AMeV and doses of 20, 30 and 40 Gy), unaffected by the addition of butyrate (control cultures). **(A1-A3)** The effects of added butyrate on wild-type cell growth. Individual batch cultures were carried out in chemically defined P2-medium (performed in serum bottles) containing glucose (approximately 42 g·L^-1^) and supplemented with 3.6 (A1), 7.2 (A2) and 10.8 g·L^-1^ (A3) of butyric acid. **(B1-B3, C1-C3, D1-D3)** The effect of added butyrate on the growth of the irradiated cells (energy 114 AMeV and doses of 20, 30 and 40 Gy). Individual batch cultures were carried out in chemically defined P2-medium (performed in serum bottles) containing glucose (approximately 42 g·L^-1^) and supplemented with 3.6 (X1), 7.2 (X2) and 10.8 g·L^-1^ (X3) of butyric acid. **(E1-E3, F1-F3, G1-G3)** The effects of added butyrate on the growth of the irradiated cells (energy 114 AMeV and doses of 20, 30 and 40 Gy). Individual batch cultures were carried out in chemically defined P2-medium (performed in serum bottles) containing glucose (approximately 42 g·L^-1^) and supplemented with 3.6 (X1), 7.2 (X2) and 10.8 g·L^-1^ (X3) of butyric acid.

*C. tyrobutyricum* ATCC 25755 uses glucose or xylose as a carbon and energy source. The monosaccharide is transported into the cell via a phosphoenolpyruvate-dependent phosphotransferase uptake system. Thereafter, glucose or xylose is metabolized via glycolysis [72,73], which exhibits an insignificant pH dependence in the range pH 7 to pH 5.5. However, the fermentations were stopped when glucose or xylose was no longer consumed by the cells because of inhibition by butyrate. To further investigate the specific effect of irradiation on the cell growth profiles (based on measurements of the optical density (OD) of cell suspension at 600 nm), individual batch cultures were carried out in chemically defined P2-medium (performed in serum bottles) containing 42 g·L^-1^ of glucose and supplemented with 3.6, 7.2 and 10.8 g·L^-1^ of butyric acid. The pH of the culture of *C. tyrobutyricum* ATCC 25755 (Figure 2A, control) dropped to around 4.8 (ΔpH of 1.4, from pH 6.2) compared with when it was supplemented with 3.6 g·L^-1^ of butyric acid (Figure 2A1), 7.2 g·L^-1^ of butyric acid (Figure 2A2) and 10.8 g·L^-1^ of butyric acid (Figure 2A3), the corresponding pH values were about 6.0 (ΔpH of 0.5 starting from 6.5), 6.1 (ΔpH of 0.3 starting from 6.4) and 5.9 (ΔpH of 0.5 starting from 6.4), respectively. However, when the culture was irradiated with 68 AMeV at a dose of 40 Gy (Figure 2D, control), the pH dropped to around 4.8 (ΔpH of 1.7 starting from 6.5) while at a dose of 40 Gy (supplemented with 3.6 g·L^-1^ of butyric acid) (Figure 2D1), a dose of 40 Gy (supplemented with 7.2 g·L^-1^ of butyric acid) (Figure 2D2) and a dose of 40 Gy (supplemented with 10.8 g·L^-1^ of butyric acid) (Figure 2D3), the pH values were about 4.6 (ΔpH of 1.6 starting from 6.2), 4.8 (ΔpH of 1.4 starting from 6.2) and 5.9 (ΔpH of 0.3 starting from 6.2), respectively. When the culture was irradiated at 114 AMeV and a dose of 40 Gy (Figure 2G, control), the pH dropped to around 5.7 (ΔpH of 0.6 starting from 6.3) while at a dose of 40 Gy (supplemented with 3.6 g·L^-1^ of butyric acid) (Figure 2G1), a dose of 40 Gy (supplemented with 7.2 g·L^-1^ of butyric acid) (Figure 2G2) and a dose of 40 Gy (supplemented with 10.8 g·L^-1^ of butyric acid) (Figure 2G3), the pH values were about 5.7 (ΔpH of 0.6 starting from 6.3), 5.4 (ΔpH of 0.9 starting from 6.3) and 5.6 (ΔpH of 0.7 starting from 6.3), respectively. When the culture was irradiated at 68 AMeV and a dose of 20 Gy (supplemented with 7.2 g·L^-1^ of butyric acid) (Figure 2B2), the pH dropped to around 4.4 (ΔpH of 0.9 starting from 6.3) while at a dose of 30 Gy (supplemented with 7.2 g·L^-1^ of butyric acid) (Figure 2C2) and a dose of 40 Gy (supplemented with 7.2 g·L^-1^ of butyric acid) (Figure 2D2), the pH values were about 4.6 (ΔpH of 1.7 starting from 6.3) and 4.8 (ΔpH of 1.5 starting from 6.3), respectively. When the culture was irradiated at 114 AMeV and a dose of 40 Gy (supplemented with 10.8 g·L^-1^ of butyric acid) (Figure 2E3), the pH decreased to 5.9 (ΔpH of 0.4 starting from 6.3) while at a dose of 30 Gy (supplemented with 10.8 g·L^-1^ of butyric acid) (Figure 2F3) and a dose of 40 Gy (supplemented with 10.8 g·L^-1^ of butyric acid) (Figure 2G3), the pH values were about 6.0 (ΔpH of 0.3 starting from 6.3) and 5.8 (ΔpH of 0.5 starting from 6.3), respectively.

These differences in pH regulate the temporal switch associated with solvent formation for each irradiated strain. This suggests that the wild-type and irradiated strains exhibited a biphasic metabolic pattern strongly influenced by the pH of the medium. As a general trend, the cells initially consumed glucose to support growth and produce and excrete organic acids (butyrate and acetate) as primary metabolites (acidogenesis), which caused a decrease in the medium pH when they accumulated to certain levels. This increase in broth acidity shifted the formation of acids towards the production of solvents when the culture reached the stationary phase of cell growth (solventogenesis). At high pH, organic acids are mainly formed, whereas at low pH, solvent production is stimulated. As expected, the nature of the metabolic shift and the kinetic pattern of solvent formation were strain dependent, given that irradiated strains exhibited their own intrinsic genetic and metabolic characteristics. Butyric acid has previously been reported to inhibit cell growth [74]. The results showed that in the wild-type strains, there was a gradual inhibition of cell growth, with no realistic growth observed at butyric acid concentrations above 3.6 g·L^-1^. However, in the irradiated strains, there was no gradual inhibition of cell growth, and no realistic growth was observed for butyric acid concentrations above 10.8 g·L^-1^.

**Figure 3:**
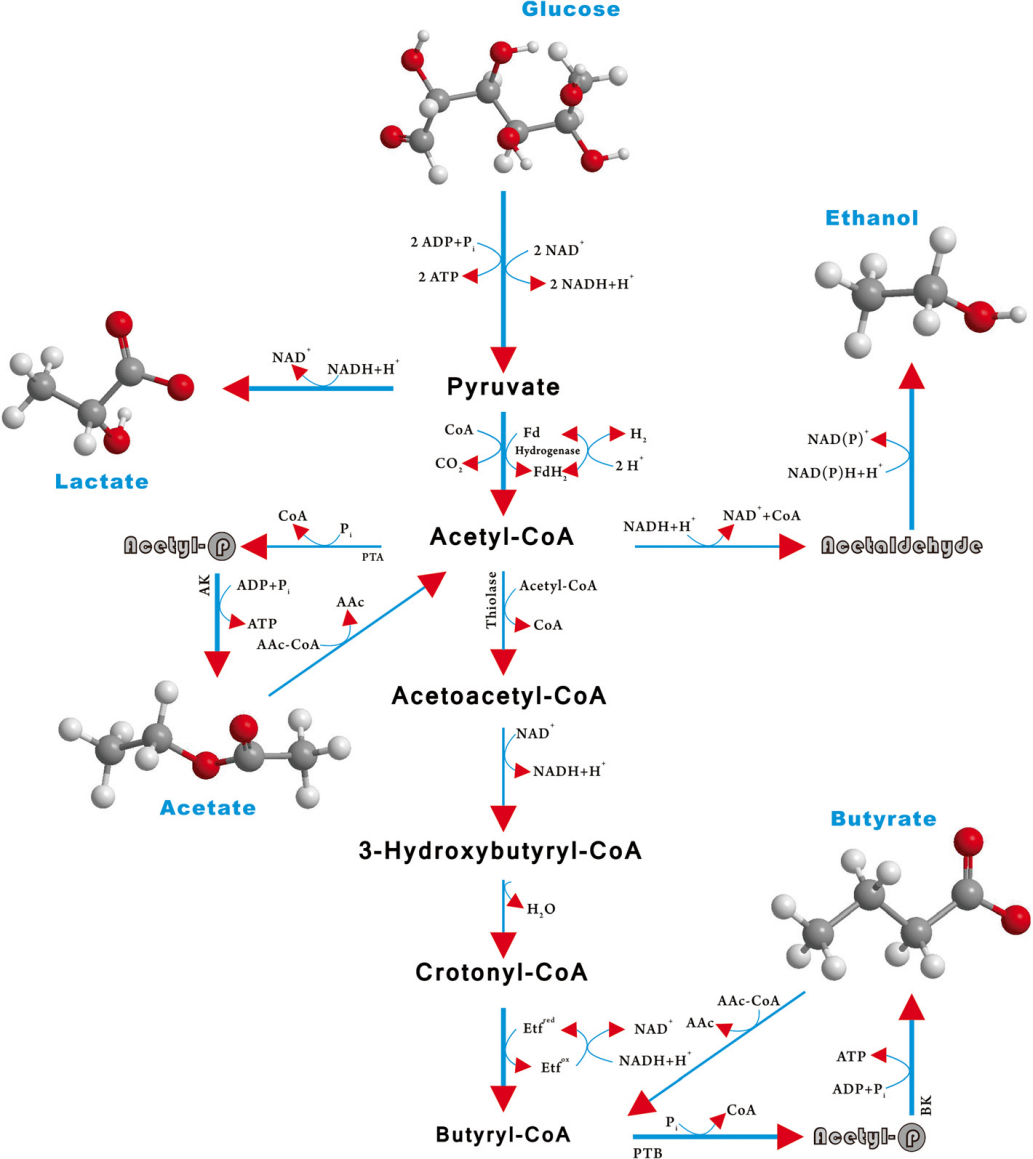
**Metabolic pathways of glucose metabolism in *Clostridium tyrobutyricum*.** Enzymes are indicated by letters as follows: lactate dehydrogenase; pyruvate-ferredoxin oxidoreductase; NADH-ferredoxin oxidoreductase; NADPH-ferredoxin oxidoreductase; hydrogenase; phosphate acetyltransferase (phosphotransacetylase); acetate kinase; acetaldehyde dehydrogenase; ethanol dehydrogenase; thiolase (acetyl-CoA acetyltransferase); acetoacetyl-CoA; acetate/butyrate:CoA transferase; acetoacetate decarboxylase; 3-hydroxylbutyryl-CoA dehydrogenase; crotonase; butyryl-CoA dehydrogenase; phosphate butyltransferase (phosphobutyrylase); butyrate kinase.

To examine in more detail the effect of added butyrate, the cell growth profiles (based on OD measurements) for the wild-type strains and irradiated strains were compared (Figure 2A1-G3) during the first 54 h of fermentation. Interestingly, the butyric acid tolerance of strains was greatly enhanced when the energy and dose of ^12^C^6+^-ion irradiation was increased. The metabolic pathways of glucose metabolism in *C. tyrobutyricum* ATCC 25755 are shown in Figure 3. The acetyl-CoA, acetoacetyl-CoA and butyryl-CoA are three key intermediates, and are of particular interest for fermentation with respect to the potential for formation of different products during acidogenesis or solventogenesis. These intermediates are important branch points that direct the metabolic flow either to acid or to solvent formation. As the last key intermediate, butyryl-CoA initiates the formation of butyric acid/butyrate. Butyrate is produced by the sequential activities of PTB, and BK [75,76]. Both enzymes are most active during acidogenesis and their specific activities decline during solventogenesis, two-fold for PTB and six-fold for Buk [76,77]. Usually a strong pH-dependent activity with an *in vitro* optimum at acidogenic pH levels of pH 5.5 (optimal around pH 4.7) and an *in vivo* (endogenous) pH greater than 5.5 are required to induce solventogenesis. Yet, a comparative analysis of these plots clearly revealed one major cluster composed of the strains irradiated at 68 AMeV and 40 Gy and the strains irradiated at 114 AMeV and doses of 30 and 40 Gy. The two groups showed a very similar overall tolerance to increasing butyrate concentrations when compared with the wild-type bacteria.

### Effect of ^12^C^6+^ ion irradiation on butyric acid production

**Figure 4:**
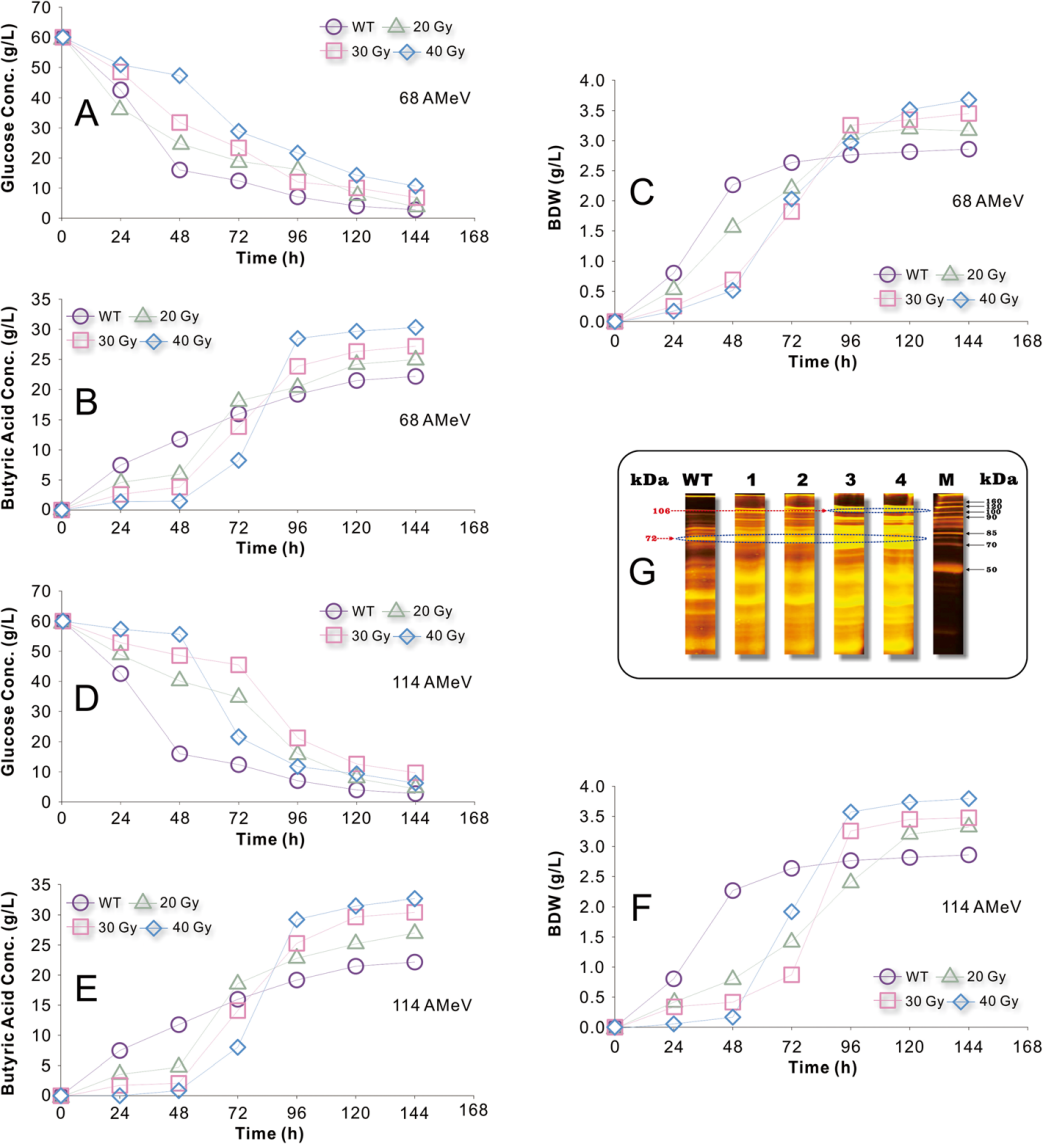
**Comparison of acid and biomass dry weight production of wild-type and irradiated *C. tyrobutyricum *grown on glucose (containing 60 g·L**^**-1**^**) in clostridial growth medium at 37°C at pH 5.5 and 6.0. (A-C)** Impact of ^12^C^6+^-ion irradiation (energy 68 AMeV and doses of 20, 30 and 40 Gy) on glucose concentration, butyric acid production and biomass dry weight. **(D-F)** Impact of ^12^C^6+^-ion irradiation (energy 114 AMeV and doses of 20, 30 and 40 Gy) on glucose concentration, butyric acid production and biomass dry weight. **(G)** SDS-PAGE of cellular proteins from *C. tyrobutyricum*. Lane WT: wild type; lane 1: cells irradiated at 68 AMeV and a dose of 30 Gy; lane 2: cells irradiated at 68 AMeV and a dose of 40 Gy; lane 3: cells irradiated at 114 AMeV and a dose of 30 Gy; lane 4: wild type and cells irradiated at 114 AMeV and a dose of 40 Gy; lane MW: protein molecular weight markers.

The butyric acid production of the irradiated strains was greatly improved in terms of both the final product concentration and yield compared with the wild-type strain as shown in Figure 4B,E. The non-irradiated (wild-type strain, control) *C. tyrobutyricum* culture inoculated into glucose-minimal media started to consume sugar almost immediately, with butyric acid production beginning 12 to 18 h later (Figure 4A,B). The same control culture inoculated into clostridial growth medium (CGM) containing 60 g·L^-1^ glucose required over 96 h to acclimate despite the fact that the irradiated strains and wild-type strain fermentations were tested under the same conditions. The extended period of minimal metabolism and productivity is because the radiation (different parameters) caused a delay in the log phase of cellular growth (Figure 4C,F). The butyric acid tolerance of the irradiated strains was greatly enhanced, allowing them to produce more butyric acid, resulting in complete glucose utilization and production of over 32 g·L^-1^ of butyric acid and similar levels of cell biomass. Furthermore, the butyric acid/control ratio increased from 1.0 for the wild-type strain to 1.52 for the strains irradiated at 114 AMeV and 40 Gy, 1.37 for the strains irradiated at 114 AMeV and 30 Gy, 1.41 for the strains irradiated at 68 AMeV and 40 Gy, and 1.31 for the strains irradiated at 68 AMeV and 30 Gy. This trend indicates that the carbon and energy flux were redistributed in the metabolic pathways of the irradiated strains, which also resulted in significant changes in the production of various fermentation products. It should be noted that the acetic acid production (data not shown) leveled off much sooner than butyrate/butyric during the fermentation. The fermentations stopped when glucose was no longer consumed by the cells because of an accumulation of organic acids and waste products in the broth, which caused inhibition of cell growth and other activities. However, the irradiated strains were more tolerant to butyric acid, as indicated by the much higher final butyrate concentration attained in the fermentations with these irradiated strains compared with the wild-type. This is not altogether surprising; as shown in Figure 3, the increased butyric acid tolerance of the irradiated strains may also be attributed to the reduced flux through the butyrate PTA/AK pathway. Since the irradiated strains were no longer dependent on the PTA/AK pathway for energy production and survival, they became less sensitive to butyric acid inhibition [11,18].

Induction of the *ack* and *pta* genes, which encode enzymes associated with the acetate formation pathway, significantly improve butyric acid production [76-78]. To better understand the fermentation kinetics of glucose metabolism after exposure of *C. tyrobutyricum* to ^12^C^6+^-ion irradiation and the resulting damage to the *ack* and *pta* genes, the protein expression of wild-type and irradiated strains were studied and compared. Figure 4G shows the results from SDS-PAGE. Analysis confirmed the expression of the protein (molecular weight, approximately 85 kDa) in four irradiated strains, with the highest protein expression level in lane 4. The amount of an approximately 106 kDa protein was much higher for the strain irradiated at 114 AMeV and 40 Gy than the wild-type strain. AK and PTA from several microorganisms have been characterized, but the results showed large variations in their molecular weight [77]. Enzyme activity assays were thus carried out to further study the roles of AK, PTA and PTB in the acid-forming pathways (Figure 3). The metabolic selectivity in *C. tyrobutyricum* is influenced by growth stage, with exponentially growing cultures producing both butyric and acetic acids, whereas slower stationary growth rates tend to produce butyric acid [3,78,79]. As such, during the log phase growth of each batch, culture samples were removed and analyzed for the activities of PTA, PTB and AK in the irradiated and wild-type strains. The specific enzyme activities for PTA, PTB and AK in the irradiated strains (different physical parameters) were assayed and their relative activities were compared with those of the wild-type strain. The AK activity was reduced by approximately 47% for the strains irradiated at 114 AMeV and 40 Gy, 31% for the strains irradiated at 114 AMeV and 30 Gy and 26% for the strains irradiated at 68 AMeV and 40 Gy. Compared with the wild-type strains, the strains irradiated at 114 AMeV and 40 Gy had a lower AK activity (47%) but unexpectedly higher PTA activity (129%), although similar PTB activities. Because the strains irradiated at 114 AMeV had much lower AK activity, the PTA-AK pathway would have been impaired and, thus, they produced more butyrate (60 g·L^-1^) from glucose than the wild-type strains. As mentioned earlier, these enhancements and improvements can be attributed to an enhanced tolerance to butyrate inhibition and to some extent the reduced carbon flux through the PTA-AK pathway as evidenced by the increased butyrate/acetate ratio in the irradiated strains.

### Effect of ^12^C^6+^ irradiation on acid yield and growth of *C. tyrobutyricum*

**Figure 5:**
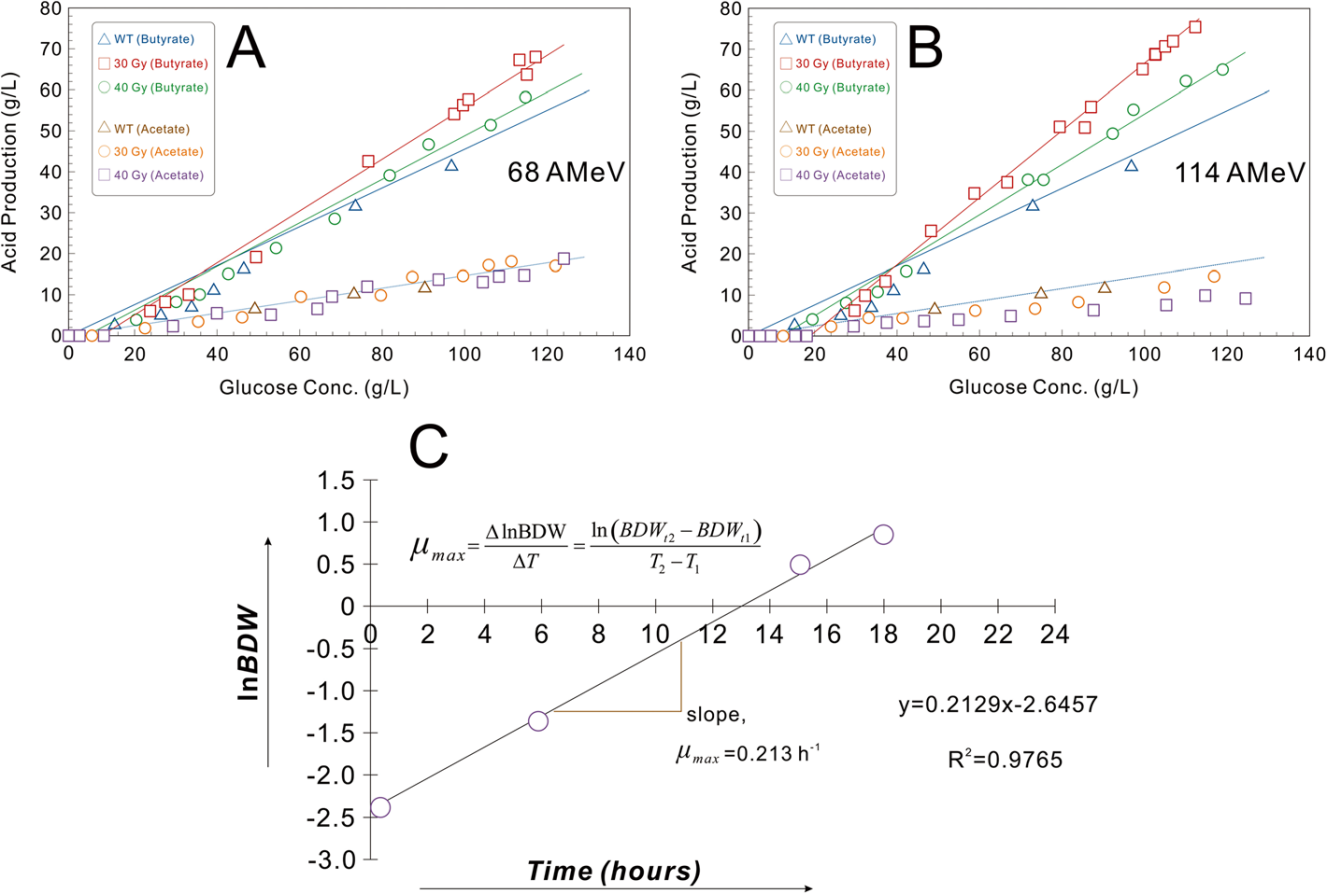
**Comparison of acid production for the wild-type and irradiated *C. tyrobutyricum *cells. (A)**The production of butyric acid and acetic acid were estimated from the slopes of the linear plots for the wild-type cells and the cells irradiated at 68 AMeV and doses of 30 and 40 Gy. **(B)** The production of butyric acid and acetic acid were estimated from the slopes of the linear plots for the wild-type cells and the cells irradiated at 114 AMeV and doses of 30 and 40 Gy. **(C)** Linearization (integration) of the kinetic growth profiles of biomass dry weight over time using a natural logarithm transformation. The maximal specific growth rates for the wild-type and the irradiated cells were calculated according to the example given for the cells irradiated at 114 AMeV and a dose of 40 Gy grown in Clostridium growth medium containing 60 g·L^-1^ of glucose. BDW, biomass dry weight.

An experiment was conducted in fermentation mode using glucose as the primary carbon source in order to determine the butyrate production capacity of *C. tyrobutyricum* ATCC 25755 after irradiation. As can be seen in Figure 5A,B, the butyric acid yield from glucose increased significantly, from 0.43 g·g^-1^ for the wild-type strains to 0.56 g·g^-1^ for the strain irradiated at 68 AMeV and a dose of 30 Gy, 0.59 g·g^-1^ for the strain irradiated at 68 AMeV and a dose of 40 Gy, 0.63 g·g^-1^ for the strain irradiated at 114 AMeV and a dose of 30 Gy, and 0.66 g·g^-1^ for the strain irradiated at 114 AMeV and a dose of 40 Gy. It is of note that the butyrate yield for the strain irradiated at 114 AMeV and a dose of 40 Gy would have been higher (>0.66 g·g^-1^) if the glucose consumption during the lag phase was neglected. The acetic acid produced by the strain irradiated at 68 AMeV and doses of 30 and 40 Gy were similar to that from the wild type. However, the acetic acid produced by the strain irradiated at 114 AMeV and doses of 30 and 40 Gy decreased compared with that of the wild type. As shown in Figure 5B, the acetic acid yield from glucose also decreased significantly, from approximately 0.11 g·g^-1^ for the wild-type strain to around 0.08 g·g^-1^ for the strain irradiated at 114 AMeV and 30 Gy, and around 0.07 g·g^-1^ for the strain irradiated at 114 AMeV and 40 Gy. Nevertheless, the butyrate/acetate ratio (g/g) increased from 3.99 for the wild-type strain to 5.82 for the irradiated strains, a clear indication that the metabolic pathways in the irradiated strains were shifted to favor butyric acid production over acetic acid production. As shown in Figure 3, since AK and PTA activities were significantly reduced in the irradiated strains, more pyruvate must have been catabolized through the butyrate-producing pathway, leading to higher butyrate yields from glucose. In addition, the butyric acid could have also promoted an earlier shift to the acid-producing pathway, which might be reflected in a slower growth rate. For the same reason, the irradiated samples suffered from a slower growth rate because less ATP was produced from the acetate-producing (PTA-AK) pathway, which normally can generate more ATP per mole of glucose metabolized than the butyrate-producing (PTB-BK) pathway [36].

A plot of *μ*_*max*_ was then determined at high concentrations of initial glucose (40, 60, 80 and 120 g·L^-1^) by fitting the fermentation data to the predictions from the model simulation. Linearization (integration) of the kinetic growth profiles of biomass dry weight (BDW) over time were achieved by using the natural logarithm transformation:

$$x\left(t\right)=x_{0}\mathrm{\cdot exp}\left(\mu \right)\to \mathrm{lnx}\left(t\right)=\mathrm{\mu \cdot t}+\ln x_{0}\to y=m+b\left(\mathrm{straight}\;\mathrm{line}\;\mathrm{equation}\right).$$

Where *x(t)* = BDW concentration at every time *x*_*0*_; t = initial BDW concentration; *μ*_*max*_ = maximum specific growth rate (h^-1^); and the specific growth rate is *μ* = (1/*x*(*t*)) *·* (*dx*/*dt*). For simplification purposes, it was assumed that all bacteria followed the exponential law of cell growth in a batch culture according to a first-order kinetic model [80-82]. The specific growth rate of cells, or increase in cell mass over time, represents a shift in selectivity at different growth rates, which has a significant impact on the fermentation process [83]. Rapid cell growth has a higher energy demand and preferentially produces acetic acid. At low growth rates, the production of butyric acid is favored over acetic acid [84]. For continuous fermentation, the production of butyrate/butyric acid is higher when *μ* is lower. When *μ* tends to zero, an oscillation in productivity occurs [85-88]. These equations allow a comparison of the growth rate of batch and continuous systems within the wild-type and the radiated strains.

The model is not media-independent: the media used as described above affects both the cell growth rate and the quantities of butyrate/butyric acid produced, and differing glucose consumption profiles would produce different results. To better quantify the optimal glucose concentration for the cell growth, the maximal specific growth rates were determined for the wild-type and irradiated strains from kinetic data taken from the exponential growth phase and plotted against the concentration of added glucose. As can be seen in Figure 5C, the maximal specific growth rates for the strains irradiated at 114 AMeV and a dose of 40 Gy were calculated according to the example where the strain was grown in CGM medium containing 60 g·L^-1^ of glucose. The best linear range of data points was chosen that corresponded to the exponential growth phase of the strain. In some cases, where the minimum requirement of three experimental data points was not satisfied, an alternative expression was utilized that accounted only for two extreme points (at the beginning and at the end of the exponential phase). The straight line slope (m = *μ*_max_) gives the maximal specific growth rate (0.213 h^-1^). The unary linear regression model (*y* = 0.2129*x* - 2.6457) had an adjusted determination coefficient of *R*^*2*^ = 0.9765, indicating that all data points were included on the line of best fit and no data points varied from this line. Additionally, each specific growth rate was estimated from the slope of the corresponding semi-logarithmic plot of the BDW versus time. Error bars are expressed in terms of the standard deviation (SD) obtained from calculations of each independent fermentation replicate for the irradiated strains and wild type (the original data is not shown). The results demonstrate that these irradiated strains had a significantly lower specific growth rate (*μ* = 0.38 ±0.03 to 0.21 ±0.02 h^-1^) as compared with the wild type (*μ* = 0.38 to 0.42 h^-1^). The use of ^12^C^6+^-ion irradiation at 68 AMeV, 20 to 40 Gy and 10^6^ to 10^8^ ions·pulse^-1^ resulted in a particularly long lag phases of 10, 12 and 16 h, respectively. By comparison, the use of ^12^C^6+^-ion irradiation at 114 AMeV, 20 to 40 Gy and 10^6^ to 10^8^ ions·pulse^-1^ resulted in lag phases of 12, 18 and 24 h, respectively. These longer lag phases may be partially attributed to the different radiation parameters and the low density inoculation amount used in the fermentation. The lower specific growth rate for the irradiated cells may be a result of the metabolic burden placed on cells as a result of the lower amount of energy generated by glucose metabolism because of damage induced at higher energy and doses. Compared with the wild-type strains, the 20 and 30 Gy irradiated strains at 68 AMeV had similar growth and glucose consumption profiles, with an almost identical specific growth rate of *μ* = 0.42 ±0.03 h^-1^, whereas the 30 and 40 Gy irradiated strains at 114 AMeV showed a significantly longer lag phase, slower glucose consumption, and a much lower specific growth rate of *μ* = 0.26 ±0.03 h^-1^ (30 Gy) and *μ* = 0.21 ±0.02 h^-1^ (40 Gy).

As noted earlier, acetate is synthesized via PTA and AK reactions with the latter reaction providing ATP (Figure 3). For the biosynthesis of butyrate, two molecules of acetyl-CoA are condensed to acetoacetyl-CoA, followed by a reduction to butyryl-CoA, which is then converted to butyrate via PTB and BK reactions with ATP generation. The lower specific growth rate for the irradiated strains (energy 114 AMeV and doses of 30 and 40 Gy) can be attributed to the metabolic burden on cells caused by less energy (ATP) generation during glucose metabolism because of irradiation damage of *ack* and *pta*. The BDW from glucose for the irradiated strains also varied from the wild-type strains. The plot of BDW versus time and specific growth rate of the irradiated strains indicated that the carbon and energy flux were redistributed throughout the metabolic pathways of these strains, which also resulted in significant changes in acid production of fermentation products.

## Conclusions

Examination of the effect of ^12^C^6+^ heavy ion irradiation on the fermentation performance of acid-producing *C. tyrobutyricum* ATCC 25755^T^ strain was performed. Following irradiation, the presence of increasing butyrate concentrations in the media resulted in a gradual inhibition of cell growth for butyric acid concentrations above 10.8 g·L^-1^, with no realistic growth observed for butyric acid concentrations above 3.6 g·L^-1^ for the wild-type strains during the first 54 h of fermentation. The irradiated strains were shown to be tolerant to butyric acid inhibition. In addition, the specific growth rate decreased by 50% and the final concentration of butyrate increased by 68% when the strains were irradiated at 114 AMeV and a dose of 40 Gy compared with the wild-type strains. This study demonstrates that butyric acid production from glucose can be significantly improved and enhanced by using ^12^C^6+^ heavy ion irradiated *C. tyrobutyricum*. Future work includes adaptation of the concept of acetone-butanol-ethanol fermentation for use in a continuous fibrous-bed bioreactor by using ^12^C^6+^ heavy ion irradiated *Clostridium* sp*.* to improve and enhance the bioproduction of biofuels. Improvement in the production of biofuels should ultimately make them more competitive in the marketplace.

## Methods

### Bacterial cultures and medium

To test butyrate/butyric acid production by various strains, a rich P2 medium containing 60 g·L^-1^ glucose, 3.6 g·L^-1^ yeast extract, 2.7 g·L^-1^ peptone, 3.2 g·L^-1^ K_2_HPO_4_, 3.2 g·L^-1^ KH_2_PO_4_, 0.2 g·L^-1^ MgSO_4_, 0.2 g·L^-1^ MnSO_4_, 0.02 g·L^-1^ FeSO_4_, 0.02 g·L^-1^ NaCl, 1.5 g·L^-1^ yeast extract (Difco, Detroit, MI, USA), 2.5 g·L^-1^ ammonium acetate, 0.0005 g·L^-1^ p-aminobenzoate, 0.0005 g·L^-1^ thiamin, 0.00005 g·L^-1^ biotin and 35 μg·mL^-1^ thiamphenicol was used. To test butyrate/butyric acid production from different substrates, the same rich P2 medium containing 30 g·L^-1^ of a different carbon source (gluconate, glucose, mannose, sorbitol, mannitol, xylose or glycerol) was used. Unless otherwise noted, the fermentation was carried out in serum bottles, each containing 40 mL of the medium and inoculated with 1% (v/v) of an overnight culture in reinforced clostridial medium (Difco) at 37°C and 250 rpm. The pH was kept between 5.0 and 6.5 by adding NaOH solution twice a day.

### Experimental setup, irradiation and MTT assay

The experiment was performed at the Cancer Therapy Terminal of the HIRF). The upgraded accelerator system of HIRFL consists of Sector Focus Cyclotron, Separated Sector Cyclotron, the CSRm and the experimental Cooling Storage Ring. High-energy ^12^C^6+^ ions with energy of 207 AMeV and 162 AMeV were extracted by CSRm. The energies of 68 AMeV and 114 AMeV were obtained by adding the absorbers (water) and calibrated by using the LISE program^a^, and the corresponding uncertainty of the energies was no more than 0.22% [89-91]. The extraction time of the carbon ions (about 10^6^ to 10^8^ ions·pulse^-1^) was about 3 s and the priming dose 10 to 50 Gy. Dose rates were up to 1.0 Gy·min^-1^. For irradiation experiments, strains cell were grown in flasks (15 cm^2^) to reach 90% confluence and they were completely filled with Dulbecco's modified Eagle's medium to avoid artifacts by irradiation through air layers [92]. The protocol is adapted from literature methods [93]. Briefly, Dulbecco's modified Eagle's medium was supplemented with 100 μL of MTT reagent (c = 0.5 g·L^-1^) to each well and incubated for 30 min at 37°C. The MTT solution was then removed. After addition of 180 μL of dimethyl sulfoxide, the plates were incubated for 15 min at 37°C to dissolve the formazan crystals [94]. Absorbance readings of dimethyl sulfoxide extracts were performed at 560 nm with reference of 690 nm using a Tecan Infinite F 200 microplate reader (Crailsheim, Germany). Treated cells were harvested the next day using trypsinization, counted and a specific number of strains cell (600 and 300 strains cell) were plated in petri dishes in triplicate for clonogenic assay. The multiple MTT assay was performed using 96-well-plates with 3,000 or 6,000 cells per well. The survival fraction was calculated from the following equation:

(1) $$\mathrm{Survival}=2^{-\frac{t_{\mathrm{delay}}}{t_{\mathrm{doublingtime}}}}$$

in which t_doubling time_ is the time period required for a quantity of cells to double and t_delay_ is the time period to reach specific absorption value of control versus irradiated cells.

### Biomass concentration and specific growth rate

After irradiation, cultures were inoculated with 0.9% (v/v) of nonsporulated preculture (OD 600_nm_ = 2 on various nutritional medium) and incubated at 37°C and 125 rpm with CGM containing glucose in 1 L bottles. Growth was tracked by monitoring light scattering at 600_nm_ with a SmartSpec™ 3000 spectrophotometer over a period of every 24 h for 5 days. Growth kinetics experiments were determined on a graph representing L*n(OD 600*_*nm*_*)* = *f(t)*. Doubling times (*d*) were calculated during the exponential phase according to the formula:

$$n=\left(\mathrm{Ln}\left(\mathrm{OD}t_{2}\right)-\mathrm{Ln}\left(\mathrm{OD}t_{1}\right)/\mathrm{Ln}\left(2\right)\;\mathrm{and}\;d=t_{2}-t_{1}/n\right)$$

where n represents the number of generations. Cultivation performance was in general judged by the yield of the butyrate/butyric acid production. As units, the yield per volume of cultivation broth (grams per 1,000 mL) and specific yield per biomass cell weight (grams per 1,000 mL) were measured at the end of cultivation. For determination of specific productivity, the growth curve of the *C. tyrobutyricum* ATCC 25755 strains, using BDW as biomass, was integrated, yielding the biomass dry weight integral (BDWI).

(2) $$\mathrm{BDW}I_{t2}=\mathrm{BDW}I_{t1}+\left[\frac{\mathrm{BD}W_{t1}+\mathrm{BD}W_{t2}}{2}\left(t_{2}-t_{1}\right)\right]$$

The BDW was determined following the protocol given by Wucherpfennig [95] with modifications. Culture samples (10 mL) were taken in 20 mL centrifuge tubes. The cells were measured gravimetrically by filtering (Nalgene 300–4100) a defined amount of biomass suspension through a pre-dried and pre-weighted suction filter (Filter Paper, Grade 392, Anugrah Niaga Mandiri, Jakarta, Indonesia). The filter was rinsed several times with deionized water to remove medium components from the biomass [96] and dried at 105°C to a constant weigh for 48 h. The BDW concentration (grams per 1,000 mL) was calculated as the difference between the weight of the filter with and without dried biomass divided by the sample volume. The BDW data points from the logarithmic growth phase were plotted on a semi-log graph to locate the period in which the culture experienced the fastest growth. These points were then used in the following equation:

(3) $$\mu_{\text{max}}\left(\frac{1}{h}\right)=\frac{\Delta \mathrm{lnBDW}}{\mathrm{\Delta T}}=\frac{\ln\left(\mathrm{BD}W_{t2}-\mathrm{BD}W_{t1}\right)}{T_{2}-T_{1}}$$

where BDW was measured in grams per 1,000 mL and time in hours. BDW_t1_ is the first point during the fastest logarithmic growth period and BDW_t2_ is the last point. T_1_ and T_2_ are described similarly.

### Analysis by SDS-PAGE

Protein samples for SDS-PAGE were prepared from the cell extract after sonication and centrifugation. The strains cell extract (15 mL) was concentrated using four volumes of acetone (60 mL) to precipitate protein at -20°C overnight, and re-dissolved in 3.5 mL of 25 mM Tris–HCl buffer (pH 7.2), following the standard protocol (Bio-Rad**,** Shanghai, China). Protein samples, 25 μg per well, were loaded into 12.5% SDS-PAGE gel and run at 100 V for 2.5 h with PROTEAN II xi Cell (Bio-Rad) and stained following the instructions of the manufacturer [97-99].

### Cell extracts and enzyme assays

The strains were cultivated in the same rich P2 medium (100 mL) at 37°C to the exponential phase (OD_600_ = -1.5). The cells were harvested, washed and suspended in 5 mL of 25 mM Tris–HCl (pH 7.4) [100]. The suspension was then sonicated, and cell debris was removed by centrifugation. The protein content of extracts was determined by the Bradford method with bovine serum albumin as the standard [101]. All these were done under ambient conditions. For the hydrogenase activity assay, cells were suspended in 150 mM Tris–HCl buffer (pH 7.4), which also contained 15 mMATP, 10 mM MgCl_2_ and 6% (w/v) hydroxylamine hydrochloride (neutralized with potassium hydroxide), and lysed at 37°C for 30 min (100 μg·mL^-1^; Sigma, Shanghai, China) [102-104]. The reaction was initiated by adding cell extract and stopped after 15 min by adding 10% (w/v) ice-cold trichloroacetic acid. Color was then developed by adding 2.5% (w/v) FeCl_3_ in 2.0 N HCl, and the absorbance at 540 nm was measured [105,106]. One unit of enzyme activity is defined as the amount of enzyme that produces 1 μmol of hydroxamic acid per min. Phosphotransacetylase and phosphotransbutyrylase were assayed with 0.2 mM acetyl-CoA and butyryl-CoA as the enzyme substrates, respectively, in 0.1 M potassium phosphate buffer (pH 7.4) following previous methods [107,108]. The enzyme activity was monitored by following the liberation of CoA at 405 nm, and one unit of enzyme was defined as the amount of enzyme converting 1 μmol of acetyl-CoA or butyryl-CoA per minute. The specific activities of AK, BK, PTA and PTB were defined as the units of enzyme activity per milligram of total protein.

### Fermentation after irradiation

After irradiation, 100 mL of cell suspension prepared in serum bottles was inoculated into the fermentor and then allowed to grow for 3 days at 37°C, agitated at 150 rpm, and pH controlled at 6.0 by adding NH_4_OH. After about 33 to 42 h of continuous circulation, most of the cells were immobilized and no change in cell density in the medium could be identified. The spent medium in the fermentor was then replaced with fresh medium, and the recirculation rate increased to 120 mL·min^-1^ for new batch fermentation. For the main fermentation, CGM was used, containing 25 to 100 g glucose, 0.65 g K_2_HPO_4_, 0.65 g KH_2_PO_4_, 0.6 g MgSO4·7H_2_O, 0.02 g MnSO_4_·7H_2_O, 0.02 g FeSO_4_·7H_2_O, 1.5 g NaCl, 2.5 g asparagines, 6 g yeast extract and 1.5 g (NH_4_)_2_SO_4_ in 1.5 L of distilled water. Modified reinforced clostridial medium (Difco) was used for pre-culture and solid culture and contained the following ingredients per liter of distilled water: 15 g glucose, 10 g tryptose, 10 g beef extract, 3 g yeast extract, 5 g NaCl, 1 g soluble starch, 0.5 g cysteine hydrochloride and 3 g sodium acetate in 15 g·L^-1^ of agar. After *C. tyrobutyricum* 25755 was pre-cultured using reinforced clostridial medium at 37°C and pH 6.0 under anaerobic conditions, the grown cells were inoculated (10%, v/v) into CGM containing glucose or pre-treated brown algae substituted for glucose. The main fermentation was conducted in a 5 L stirred-tank fermentor (BE Marubishi, Pathumthani, Thailand) with a working volume of 1.5 L, and pH was controlled at 6.0 using 3 M NaOH solutions. Anaerobiosis was reached by sparging the fermentor medium with N_2_ gas for 5 to 10 min before inoculation and constantly stirred at 150 rpm during cultivation. A detailed description of the reactor construction has been given elsewhere [54].

### Analytical methods

Cell density was analyzed by measuring the OD of the cell suspension at 600 nm using a spectrophotometer (Thermo Electron Scientific Instruments Corp., Madison, WI USA) with a conversion of 0.42 g·L^-1^ of dry cell weight per OD unit. Dry weight of immobilized-cell biomass was determined by centrifugation of the fermentation broth at 10,000 g for 10 min, washing the sediment with distilled water, and drying at 110°C overnight. Butyric acid and acetic acid were analyzed with a GC-2014 Shimadzu gas chromatograph (Shimadzu, Columbia, MD, USA) equipped with a flame ionization detector and a 30.0 m fused silica column (0.25 mm film thickness and 0.25 mm ID, Stabilwax-DA). The gas chromatograph was operated at an injection temperature of 200°C with 1 μL of sample injected with the AOC-20i Shimadzu auto injector. Column temperature was held at 80°C for 3 min, raised to 150°C at 30°C min^-1^, and then held at 150°C for 3.7 min. A high performance liquid chromatography system was used to analyze the carbonhydrate compounds, including glucose, fructose and sucrose in the fermentation broth. The high performance liquid chromatography system consisted of an automatic injector (Agilent Technologies Inc, Beijing, China), a pump (Agilent 1100, G1311A), a Zorbax carbohydrate analysis column (250 mm × 4.6 mm, 5 μm; Agilent, USA), a column oven at 30°C (Agilent 1100, G1316A), and a refractive index detector (Agilent 1100, G1362A). The mobile phase was ethyl nitrile (ethyl nitrile/water = 75:25) at a flow rate of 1.5 mL·min^-1^.

## Endnote

^a^The LISE program is designed to predict the intensity and purity of radioactive ion beams produced by in-flight separators. It also facilitates the tuning of experiments where its results can be quickly compared to on-line data.

## Abbreviations

AK: acetate kinase; BDW: biomass dry weight; BK: butyrate kinase; CGM: clostridial growth medium; CoA: coenzyme A; CSRm: Main Cooling Storage Ring; HIRFL: Heavy ion research facility in Lanzhou; MTT: 3-(4,5-dimethylthiazol-2-yl)-2,5-diphenyltetrazolium bromide; OD: optical density; PTA: phosphotransacetylase; PTB: phosphotransbutyrylase.

## Competing interests

The authors declare that they have no competing interests.

## Authors’ contributions

XZ drafted and reviewed the work, revised it critically for important intellectual content and gave final approval of the version to be published; XHL, XHL and ZJX participated in the study design and coordination and were involved in revising the manuscript critically for important intellectual contents; JRX, MRZ, LW and WYD executed the experimental work and data analysis, executed the experimental work and drafted the manuscript. JPL assisted in the experimental design and was involved in revising the manuscript critically for important intellectual content. All authors read and approved the final version of the manuscript.
